# Comparative Transcriptome Profile Analysis of *Longissimus dorsi* Muscle Tissues From Two Goat Breeds With Different Meat Production Performance Using RNA-Seq

**DOI:** 10.3389/fgene.2020.619399

**Published:** 2021-01-13

**Authors:** Jiyuan Shen, Zhiyun Hao, Jiqing Wang, Jiang Hu, Xiu Liu, Shaobin Li, Na Ke, Yize Song, Yujie Lu, Liyan Hu, Lirong Qiao, Xinmiao Wu, Yuzhu Luo

**Affiliations:** Gansu Key Laboratory of Herbivorous Animal Biotechnology, Faculty of Animal Science and Technology, Gansu Agricultural University, Lanzhou, China

**Keywords:** RNA-Seq, skeletal muscle, differentially expressed gene, Liaoning cashmere goats, Ziwuling black goats

## Abstract

Carcass weight, meat quality and muscle components are important traits economically and they underpin most of the commercial return to goat producers. In this study, the *Longissimus dorsi* muscle tissues were collected from five Liaoning cashmere (LC) goats and five Ziwuling black (ZB) goats with phenotypic difference in carcass weight, some meat quality traits and muscle components. The histological quantitative of collagen fibers and the transcriptome profiles in the *Longissimus dorsi* muscle tissues were investigated using Masson-trichrome staining and RNA-Seq, respectively. The percentage of total collagen fibers in the *Longissimus dorsi* muscle tissues from ZB goats was less than those from LC goats, suggesting that these ZB goats had more tender meat. An average of 15,919 and 15,582 genes were found to be expressed in *Longissimus dorsi* muscle tissues from LC and ZB goats, respectively. Compared to LC goats, the expression levels of 78 genes were up-regulated in ZB goats, while 133 genes were down-regulated. Gene ontology (GO) and Kyoto Encyclopedia of Genes and Genomes (KEGG) analyses revealed that the differentially expressed genes (DEGs) were significantly enriched in GO terms related to the muscle growth and development and the deposition of intramuscular fat and lipid metabolism, hippo signaling pathway and Jak-STAT signaling pathway. The results provide an improved understanding of the genetic mechanisms regulating meat production performance in goats, and will help us improve the accuracy of selection for meat traits in goats using marker-assisted selection based on these differentially expressed genes obtained.

## Introduction

As an important agricultural animal, domestic goat (Capra hircus) plays key roles in meat, fiber and milk production. Goat meat has unique characteristics of flavor and palatability. Compared to meat from other domestic animals, goat meat contains higher protein content, but lower fat and cholesterol contents (Teixeira et al., 2019). Globally, goat meat has now been accepted and recognized as an important resource of protein.

It is well known that meat yield and quality are controlled by both genetic and environment factors, so an improved understanding of molecular mechanisms that regulate skeletal muscle growth and development offers an opportunity to improve meat production and quality. In this respect, some important functional genes and signaling pathways related to meat yield and quality were found. For example, previous studies have underlined the crucial role of myostatin (*MSTN*), muscle regulatory factors (MRFs) family members (*Myf5*, *Mrf4*, *MyoD*, and *MyoG*) and Insulin-like growth factors (IGFs) family members (*IGF-1*, *IGF-2*, *IGF1R*, *IGF2R*, *IGFI*/*InsR3*, and *IGFBP1-6*) in skeletal muscle growth and development in various species, such as cattle (Kambadur et al., 1997; Muroya et al., 2002; Miyake et al., 2010), sheep (Xing et al., 2014; Siqin et al., 2017), and goats (Zhong et al., 2013; Zhan et al., 2015; Zhang et al., 2019). The wingless-type MMTV integration site family (Wnt), mitogen-activated protein kinase (MAPK) and phosphatidylinositol-3-kinase (PI3K)-Akt signaling pathways have been reported to play key roles in myogenesis, muscle growth, regeneration and differentiation in mice (Tajbakhsh et al., 1998; Liu et al., 2017), chicken (Elia et al., 2007; Abu-Elmagd et al., 2010), and human (Otto et al., 2008; Kornasio et al., 2009). In recent years, RNA-Seq has been widely used to analyze genetic mechanisms underlying skeletal muscle growth and development in pigs (Óvilo et al., 2014; Xu et al., 2018), cattle (Silva-Vignato et al., 2017; Zhang et al., 2018), and sheep (Sun et al., 2016; Cheng et al., 2020).

There are some studies that have described the muscle transcriptome of goats, but these studies have mainly been focused on goats of different ages. Wang et al. (2016) identified 6,432 differentially expressed genes in the *Longissimus thoracis* muscle tissues between fetal and juvenile Huanghuai goats, and these genes were found to be involved in fetal myogenesis, proliferation, and differentiation of muscle cells. Additionally, 111 genes were differentially expressed in *Longissimus dorsi* muscle tissues of Jianzhou Big-Eared goats between kid (2-month age), youth (9-month age), and adult (24-month age) periods and they were related to muscle development and lipid metabolism (Lin et al., 2017). However, little is known about the comparative transcriptome of muscle tissues in other goat breeds, or between different breeds of goats.

Liaoning cashmere (LC) goat is a famous local goat breed in China, used for both meat and cashmere fiber production. The live body weight of adult LC rams were 81.7 ± 4.8 kg (Zhao, 2013). Due to having high meat yield, LC goats have been widely used to improve meat yield of other goat breeds in China, including Ziwuling black (ZB) goat (Ma and Jiang, 1992). ZB goat is also a local goat breed in China. LC and ZB goats are of economic importance in the region in which they are raised. Compared to LC goats, ZB goats have smaller body size and lower meat yields. The live body weight of adult ZB rams were 34.6 ± 7.5 kg (Zhao, 2013). However, meat from ZB goats is tender, and has better favorable palatability and high contents of nutrients and flavor substances (Zhao, 2013). Sha et al. (2019) suggested that the meat from ZB goats had potential to product high-grade mutton due to its higher quality of meat traits. Despite there are relevant differences in meat production performance between LC and ZB goats, the molecular mechanism that underpins these differences remains unclear. In this study, RNA-Seq was used to compare the transcriptome profile of *Longissimus dorsi* muscle tissues between LC and ZB goats, and identify differentially expressed genes (DEGs) between the two breeds. Gene ontology (GO) enrichment and Kyoto Encyclopedia of Genes and Genomes (KEGG) pathway of DEGs were also analyzed. These results will identify relevant biological mechanisms underlying meat yield and quality in the two breeds, and will also offers an opportunity to improve meat yield and quality in goats.

## Materials and Methods

### Ethics Statement

All experimental animals were conducted according to the animal protection and use guidelines established by the Ministry of Science and Technology of the People’s Republic of China (approval number 2006-398). It was also approved by Gansu Agricultural University, Lanzhou, China.

### Experimental Animals and Sample Collection

Ten healthy nine-month-old rams were selected for the investigation at Yongfeng Goat Breeding Company in Huan County, Gansu Province, China, including five LC rams and five ZB rams. All experimental rams were raised under the same environmental conditions and had the same nutrition program. The feed ingredient included 59% corn, 13% pea, 8% oil cake of flax seed, 18% wheat bran, 1% salt, 0.5% premix, and 0.5% limestone, providing 13.52 MJ/kg digestible energy, 14.18% crude protein, 3.12% calcium, 0.41% phosphorus, and 85% dry matter. The carcass weight, meat quality and muscle components from these rams were measured after slaughtering and the detailed data are listed in Table 1 (Wang et al., 2021).

**TABLE 1:** Comparison of carcass weight, meat quality and muscle components between LC and ZB goats.

Traits	LC goats	ZB goats	p value
	(*n* = 5)	(*n* = 5)	
Carcass weight (kg)	14.10 ± 1.17	7.45 ± 1.28	2.600E-05
Muscle shear force value (N)	22.71 ± 2.63	18.11 ± 1.27	0.027
Intramuscular fat content (%)	3.23 ± 0.23	1.88 ± 0.40	0.004
Muscle drip water loss rate (%)	5.07 ± 0.51	2.92 ± 0.19	0.016
pH at 45 min post-mortem (pH_1_)	6.51 ± 0.21	6.75 ± 0.14	0.001
pH at 24 h post-mortem (pH_24_)	5.70 ± 0.10	5.86 ± 0.05	1.829E-05

When the animals were slaughtered, *Longissimus dorsi* muscle from the left half carcass, in the region of 12th and 13th ribs, were collected from individual rams to be further used in RNA-Seq and histological quantitative analysis of total collagen. The samples for RNA – Seq analysis were immediately stored in liquid nitrogen, while the samples used for histological quantitative analysis of total collagen were cut to 1.0 cm^3^ cubes and then fixed in 4% neutral paraformaldehyde.

### Histological Quantitative Analysis of Total Collagen

After fixation in 4% neutral paraformaldehyde for 24 h, the specimens were dehydrated in graded ethanol (75, 85, 95, and 100%), followed by being cleared in xylol for 5 min and embedded in paraffin at 60°C. The embedded tissue sections were cut into 5 μm of thickness using a Rotary cutting machine (Leica, Wetzlar, Germany) and then stained with Masson’s trichrome as reported by Rieppo et al. (2019).

Three different fields of view for each sample in LC and ZB goats were observed. Micrographs (400×) of Masson-trichrome staining in each sample were taken by Pannoramic 250 digital slice scanner (3DHISTECH, Budapest, Hungary). Image-Pro Plus 6.0 was used to analysis the integrated optical density and area of collagen fibers. The percentage of collagen fibers in muscle tissue was calculated, and then the difference in the proportion of collagen fibers between LC and ZB goats was tested using SPSS v17.0.

### RNA Extraction, Library Construction and Sequencing

Total RNA was extracted from caprine *Longissimus dorsi* muscle tissues using Trizol reagent kit (Invitrogen, Carlsbad, CA, United States). The concentration and integrity of RNA was assessed using a Nanodrop 2000 (Thermo Scientific, MA, United States) and Agilent 2100 Bioanalyzer (Agilent, CA, United States), respectively. Only samples with an RNA integrity number > 7 were used for constructing cDNA libraries and subsequent sequencing.

Complementary DNA (cDNA) libraries for the two groups (5 × LC goats, 5 × ZB goats) were generated using a NEBNext Ultra RNA Library Prep Kit for Illumina (New England Biolabs, MA, United States). Briefly, ribosomal RNA (rRNA) was removed from these RNA samples using a Ribo-Zero Gold rRNA Removal Kit (Illumina, CA, United States). The remaining RNA was then fragmented into 200–500 nt pieces using fragmentation buffer (New England Biolabs, MA, United States). The first strand cDNA was synthesized using random hexamers. The second-strand cDNA were subsequently synthesized using DNA polymerase I and RNase H. After end repaired and adenylation of the 3′-ends of the DNA fragments, Illumina sequencing adapters were ligated to prepare for hybridization.

In order to select cDNA fragments of the preferred ∼200 bp in length, the AMPure XP Beads (Beckman Coulter, CA, United States) were used to purify the library fragments, followed by selectively being enriched using universal PCR primer and index primer in a 12-cycles PCR reaction. The amplification products were purified and quantified using the AMPure XP Beads (1.0X) (Beckman Coulter, CA, United States) and the High Sensitivity DNA assay Kit (Agilent, CA, United States), respectively. The cDNA libraries obtained were paired-end sequenced in 28 cycles by Gene *Denovo* Biotechnology Co., Ltd (Guangzhou, China), using an Illumina HiSeq^TM^ 4000 sequencer (Illumina, CA, United States).

### Sequence Analysis and Identification of DEGs

The raw reads are stored in FASTQ file format that contains the sequence of reads and the base quality. The clean reads were obtained by removing reads containing adapters, reads with >10% unknown nucleotides and the low quality reads (those with quality scores <Q20) using fastp v0.18.0. The clean reads were further mapped to rRNA database to remove mapped rRNA reads using Bowtie2 v2.2.8. The remaining clean reads were mapped against Caprine Genome Assembly ARS1^1^ using HISAT2 v2.1.0 (Kim et al., 2015).

Gene enrichment was normalized by calculating Fragment Per Kilobase of transcript per Million mapped reads (FPKM). The genes with FPKM > 0.01 were considered to be meaningful expressed (Trapnell et al., 2010). The negative binomial distribution model of DESeq v2.0 (Love et al., 2014) was used to compare the expression levels of the samples from five LC rams with the samples from five ZB rams, and the likelihood ratio test was used for hypothesis testing. Genes with fold change > 2.0 and false discovery rate (FDR) value < 0.05 were defined as significant differentially expressed genes (DEGs).

### GO Enrichment and KEGG Pathway Analyses of the DEGs

For the functional enrichment analysis of the DEGs, the GO database^2^ was used, classifying genes into biological process (BP), molecular function (MF), and cellular component (CC) categories. Additionally, Kyoto Encyclopedia of Genes and Genomes (KEGG) database was used to perform pathway analysis of the DEGs (Kanehisa et al., 2008). The significantly enriched GO terms and KEGG pathways (*P* < 0.05) were defined using hypergeometric test.

### Validation of DEGs by Reverse Transcription-Quantitative qPCR

To verify the reliability of the RNA-Seq results, twenty of the DEGs identified were randomly selected for reverse transcription-quantitative PCR (RT-qPCR) analysis. These included ten down-regulated genes (*COL3A1*, *SPARC*, *ASIP*, *FABP3*, *CCDC80*, *LGALS1*, *PRKAG3*, *LOXL2*, LOC102178315, and *MB*) and ten up-regulated genes (*BTG2*, *IFI6*, *SOCS2*, *CDKN1A*, *MX1*, *MX2*, *PIM1*, *SDC4*, *CISH*, and *PEBP1*) in the ZB goats compared to LC goats. *GAPDH* and β*-actin* were used as housekeeping genes to normalize the expression level of these DEGs. The primer information of the above genes is listed in Table 2.

**TABLE 2:** The information of PCR primer used for RT-qPCR.

Gene	Forward (5′→ 3′)	Reverse (5′→ 3′)
*COL3A1*	CTGGCAAGGATGGAACAAGT	CTCCATAATACGGGGCAAAA
*BTG2*	AAGATGGACCCCATCATCAG	CCTGCCCAGTATCATTTGGT
*IFI6*	AAGACGGAAAAAGACGCTCA	GCCGACCAGCTCATCAAT
*SPARC*	GAAGGTGTGCAGCAACGACAAC	TTCAGTCAGCTCGGAGTCCA
*ASIP*	ATTTCCCTTCTGTCTCTAT	TGGGGTTGAGCCCGCGGC
*SOCS2*	AACTCAGTCACACAGGTTGG	TGCGAAGATTAGTTGGTCCAGC
*FABP3*	GTGAATGGGGACACAGTCATCA	GAGTTTCCCGTCAACTATTTCC
*CDKN1A*	GAGAGCGATGGAACTTCGAC	AGTGGTCCTCCTGAGACGTG
*CCDC80*	TTCCTATCCAGGTTCCGTTG	CAAATGGGCTGGTACGTCTT
*MX1*	GTCCCTGCTAACGTGGACAT	ACCAGGTTTCTCACCACGTC
*LGALS1*	GCCAGCAACCTGAATCTCAAAC	CATACCTCCGCGACACTTCCAG
*MX2*	TGCATATTTCACGGAAACCA	GAAGCAGCCAGGAATAGTGC
*PRKAG3*	GGCTTCTCAAGTTCCTGCAC	CATCAAAGCGGGAGTAGAGG
*PIM1*	CAGAGTGGATCCGCTACCAT	GCTGCCTGAAGAAAACTTGG
*LOXL2*	GAAGATGTGGGTGTGGTGTG	TTCTTAGCCGTCCAGTGCTT
*SDC4*	GTCGATCCGAGAAACTGAGG	GATCTCCAGAGCCAGACAGC
LOC10 2178315	CAAGATCACCATCACCAACG	ATCACCTCCTGGCATTTGTC
*CISH*	ACCAGTTATGCAGCCTTTGC	TCCCGAAGGTAGGAGAAGGT
*MB*	ACCTGAAGAAGCATGGCAAC	CATCAGCACCGAAGTCTGAA
*PEBP1*	GACACCCACCCAGGTTAAGA	GCCCACATAATCGGAGAGAA
*GAPDH*	ACACTGAGGACCAGGTTGTG	GACAAAGTGGTCGTTGAGGG
β*-actin*	AGCCTTCCTTCCTGGGCATGGA	GGACAGCACCGTGTTGGCGTAA

The RNA samples that were the same as those used for the RNA-Seq, were used to synthesize cDNA using SuperScript^TM^ II reverse transcriptase (Invitrogen, CA, United States). The RT-qPCR were conducted in triplicate using 2 × ChamQ SYBR qPCR Master (Vazyme, Nanjing, China) on an Applied Biosystems QuantStudio^®^ 6 Flex (Thermo Lifetech, MA, United States). The relative expression levels of these DEGs were analyzed using the 2^–ΔΔCt^ method.

## Results

### Histological Quantitative Analysis of Collagen Fibers

**FIGURE 1:**
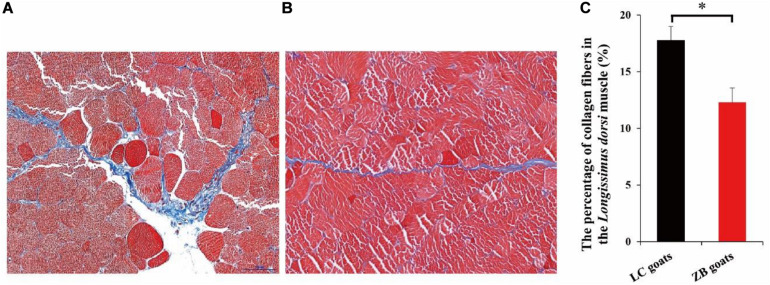
The percentage of collagen fibers in the *Longissimus dorsi* muscle (%). **(A)** The micrographs (400×) of the *Longissimus dorsi* muscle from LC rams. **(B)** The micrographs (400×) of the *Longissimus dorsi* muscle from ZB rams. The collagen fibers and muscle fibers in the *Longissimus dorsi* muscle tissues were stained with blue and red, respectively. **(C)** Comparison of the percentage of collagen fibers in the *Longissimus dorsi* muscle tissues from LC rams, with those from ZB rams; ^∗^*p* < 0.05.

In the *Longissimus dorsi* muscle tissues of both LC and ZB rams, muscle tissues mainly included collagen fibers, muscle fibers and adipose tissues, of which collagen fibers and muscle fibers were stained with blue and red in Masson’s trichrome, respectively (Figures 1A,B). It was further found that the percentage of total collagen fibers in the *Longissimus dorsi* muscle tissues from LC rams was 17.78 ± 1.21%, which was higher than those from ZB goats (12.31 ± 1.25%; *P* = 0.017) (Figure 1C).

### Summary of the RNA-Seq Data

Ten separate cDNA libraries were constructed using the *Longissimus dorsi* muscle tissues from the two goat breeds (5 × LC goats and 5 × ZB goats), all of the raw reads obtained in the study have been deposited in GenBank with accession numbers SRR13008213-SRR13008222. The summary statistics of the RNA-Seq data are shown in Table 3. On average, 88,909,052 and 87,358,060 raw reads were produced from the cDNA libraries constructed from LC and ZB goats, respectively. After filtering adaptor reads and reads with >10% unknown nucleotides and low quality reads, an average of 88,773,430 and 87,190,490 clean reads were obtained from LC and ZB goats, respectively. The correlations between the five samples within LC and ZB goats were above 0.92 and 0.93, respectively. After removing the rRNA mapped reads, the remaining clean reads were 81,987,190 and 85,024,551, respectively. Of the remaining clean reads, an average of 77,996,504 (95.13%) and 82,000,854 (96.44%) reads were well mapped to the goat reference genome assembly (ARS1), with a unique mapped rate of 90.94 and 92.78%, respectively.

**TABLE 3:** The summary of the RNA-Seq data.

Sample	Average raw reads	Average clean reads	Average remaining clean reads	Average mapped reads	Average unique reads	Average multiple reads
LC goats	88,909,052	88,773,430	81,987,190	77,996,504 (95.13%)	74,558,034 (90.94%)	3,438,470 (4.19%)
ZB goats	87,358,060	87,190,490	85,024,551	82,000,854 (96.44%)	78,888,983 (92.78%)	3,111,871 (3.66%)

Using a cut-off of >0.01 FPKM to define potentially expressed genes, a total of 15,919 and 15,582 expressed genes were detected in *Longissimus dorsi* muscle tissues from LC and ZB goats, respectively, with 15,496 genes being expressed in both breeds.

### Screening of DEGs Between ZB and LC Goats

**FIGURE 2:**
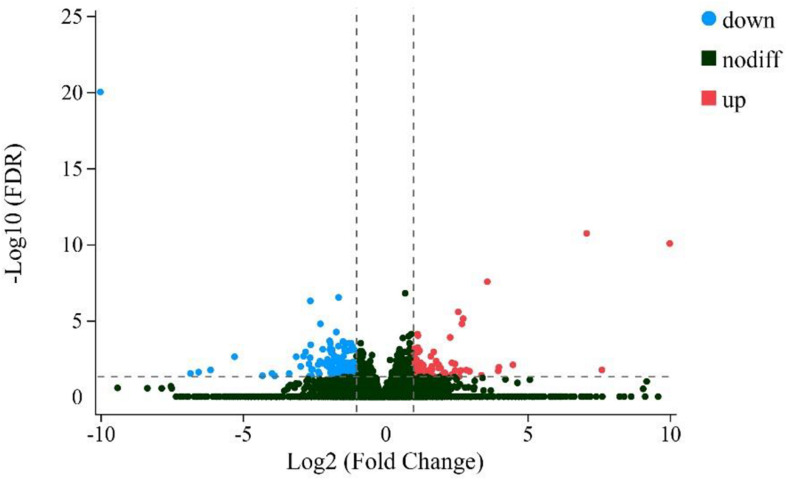
Volcano plot comparing the change in gene expression of the LC goats and ZB goats. The red and blue dots represent the up-regulated and down-regulated genes in the *Longissimus dorsi* muscle tissues of ZB goats compared to the *Longissimus dorsi* muscle tissues of LC goats, respectively (FDR < 0.05). The black dots represent genes that are not significantly different in the two caprine breeds (FDR > 0.05).

A total of 211 genes were found to be differentially expressed when comparing ZB and LC goats (FDR < 0.05) (Supplementary File 1). Of these, 78 genes had higher expression in ZB goats compared to LC goats and are therefore referred to “up-regulated,” while the remaining 133 genes had lower expression in ZB goats and are accordingly referred to “down-regulated” (Figure 2).

The top five up-regulated genes in ZB goats was uncharacterized protein C1orf43 homolog (LOC102177858), 2′-5′-oligoadenylate synthase 2-like (LOC108637852), Phosphati dylethanolamine binding protein 1 (*PEBP1*), proline-rich receptor-like protein kinase PERK8 (LOC102191280) and brain derived neurotrophic factor (*BDNF*), with 2,294.0, 196.2, 135.2, 22.5, and 16.0-fold increases in expression, respectively.

The most prominent down-regulated gene in ZB goats was Agouti signaling protein (*ASIP*) with a 1210.1-fold decrease, followed by Heat shock 70 kDa protein 1B-like (LOC102178315), 60S ribosomal protein L17-like (LOC102186409), Delta/Notch-like EGF repeat containing (*DNER*) and Hephestin like 1 (*HEPHL1*), with 114.2, 93.7, 70.0, and 39.0-fold decreases in expression, respectively.

### GO Enrichment and KEGG Pathway Analysis of the DEGs

**FIGURE 3:**
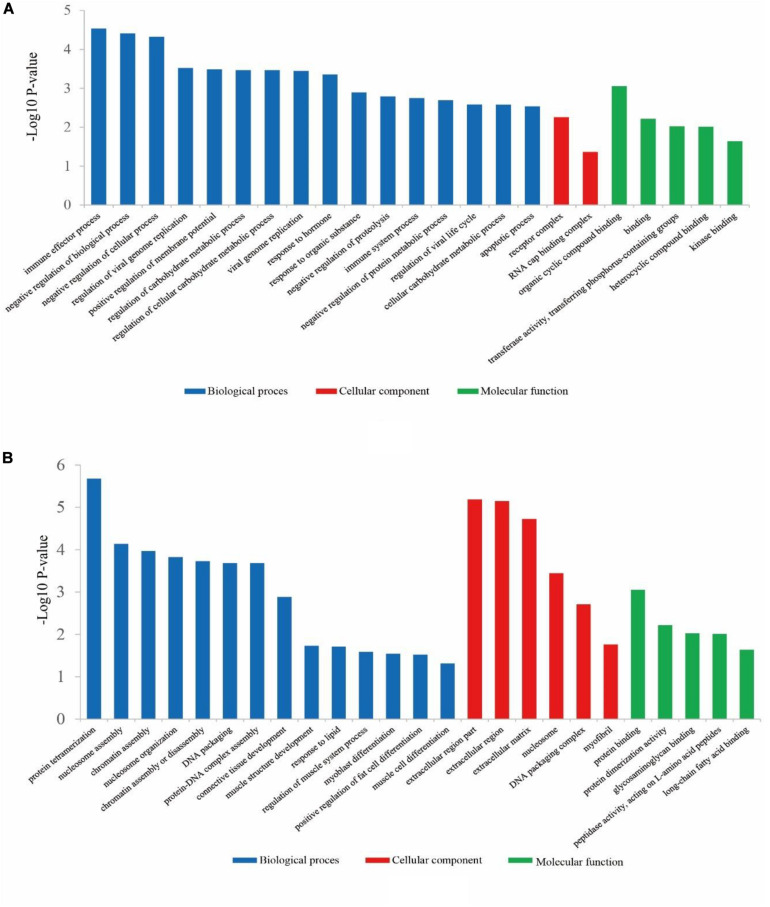
The classification of Gene Ontology (GO) terms for up-regulated genes **(A)** and down-regulated genes **(B)** in ZB goats compared to LC goats. The most enriched biological process, cellular component and molecular function GO terms are shown.

Concerning the 78 up-regulated genes in ZB goats, they were significantly enriched in 166 BP terms, 2 CC terms and 24 MF terms. The top five significant GO terms with the lowest *P*-value were immune effector process (*P* = 2.90E-05), negative regulation of biological process (*P* = 3.87E-05), negative regulation of cellular process (*P* = 4.74E-05), regulation of viral genome replication (*P* = 0.0003) and positive regulation of membrane potential (*P* = 0.00032) (Figure 3A).

Regarding the 133 down-regulated genes in ZB goats, they were significantly enriched (*P* < 0.05) in 179 BP terms, 30 CC terms and 15 MF terms. Of these GO terms, the most enriched terms were protein tetramerization (*P* = 2.10E-06), extracellular region part (*P* = 6.49E-06), extracellular region (P = 7.14E-06) and extracellular matrix (*P* = 1.88E-05). Besides these GO terms described above, some important GO terms were also found, including GO terms related to muscle growth and development (muscle structure development, regulation of muscle system process, myoblast differentiation, muscle cell differentiation, and myofibril), GO terms related to lipid metabolism (response to lipid and long-chain fatty acid binding), and the positive regulation of fat cell differentiation (Figure 3B).

**FIGURE 4:**
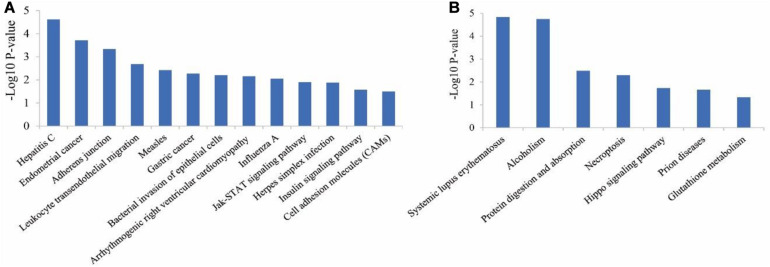
KEGG pathway analysis for the up-regulated genes **(A)** and down-regulated genes **(B)** in ZB goats compared to LC goats.

Considering the pathway enrichment analysis, for up-regulated genes in ZB goats, the most enriched pathway was hepatitis C (*P* = 2.41E-05), followed by endometrial cancer (*P* = 0.00019), adherens junction (*P* = 0.00046), leukocyte transendothelial migration (*P* = 0.00207) and measles (*P* = 0.0038). In addition, some important pathways that have been previously reported to be associated with muscle growth and development, were also identified in the study, including Jak-STAT signaling pathway (*P* = 0.013) (Figure 4A). For down-regulated genes in ZB goats, the most enriched pathway was systemic lupus erythematosus (*P* = 1.45E-05), followed by alcoholism (*P* = 1.79E-05), protein digestion and absorption (*P* = 0.0033), necroptosis (*P* = 0.0051) and hippo signaling pathway (*P* = 0.018) (Figure 4B).

### Validation of RNA-Seq Results Using RT-qPCR

**FIGURE 5:**
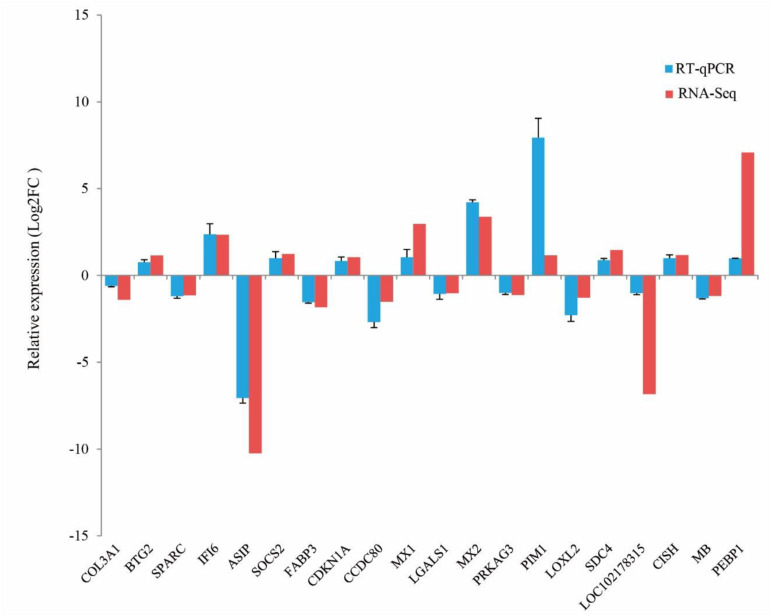
Comparison of gene expression levels obtained by RNA-Seq, with those measured using RT-qPCR for 20 randomly selected DEGs. These contained ten up-regulated genes and ten down-regulated genes in ZB goats compared to LC goats. The expression level values for the RT-qPCR were normalized by *GAPDH* and β*-actin* genes. The error bars represent the variation in the five separate goats studied per breed.

To validate the reliability of RNA-Seq data, 20 DEGs were randomly selected to perform RT-qPCR, including 10 up-regulated genes and 10 down-regulated genes in ZB goats. The results from the RT-qPCR were consistent with those obtained from the RNA-Seq data. For example, the expression levels of *BTG2*, *IFI6*, *SOCS2*, *CDKN1A*, *MX1*, *MX2*, *PIM1*, *SDC4*, *CISH*, and *PEBP1* in ZB goats were higher than those in LC goats. In contrast, compared to LC goats, the expression levels of *COL3A1*, *SPARC*, *ASIP*, *FABP3*, *CCDC80*, *LGALS1*, *PRKAG3*, *LOXL2*, LOC102178315, and *MB* were lower in ZB goats. These results confirmed the reliability and repeatability of the RNA-Seq results (Figure 5).

## Discussion

The aim of the study was to reveal molecular mechanisms that may underline the difference in meat yield, meat quality and muscle components between LC and ZB goats. Comparing to LC goats, ZB goats investigated in the study had lower muscle shear force values that result in more tender meat. Meat tenderness is regarded as the most important index of palatability trait and therefore significantly affects consumer acceptance for meat. Collagen fiber is the main component of intramuscular connective tissue in meat, and is also key factor affecting meat tenderness. It has been reported that the content of total collagen fiber in meat is highly positively correlated with the shear force value in beef (*r* = 0.95) (Dransfield et al., 2003). In this study, ZB goats had a lower proportion of total collagen in the *Longissimus dorsi* muscle tissues compared to LC goats. This was identical with the observation that ZB goats had lower muscle shear force values, resulting in more tender meat. Besides to the association with shear force values, the content of collagen fiber has been reported to be associated with carcass weight and intramuscular fat content in domestic animals. The content of collagen increased with the increase of carcass weight in bulls (Gur et al., 2003; Purslow et al., 2012). With the deposition of intramuscular fat in beef, the deposition of collagen was also enhanced (Duarte et al., 2013). Therefore, lower percentage of collagen fibers in ZB goats was related to their lower carcass weight and intramuscular fat content in the study.

With the development of RNA-Seq technology and the reduction of sequencing cost, there are increasing tendencies for the number of sequencing samples per condition. In our study, five samples per goat breed were used for RNA-Seq. Although the sample number may be a limitation in our study, the number of samples has not significant effect on the results of RNA-Seq based on the following reasons. First, the correlations between the five samples within LC and ZB goats are above 0.92 and 0.93, respectively. This indicated that there is a small variation for meat performance within a given breed and the five samples are of representative. Meanwhile, Schurch et al. (2016) found that true positive rate is largely insensitive to the number of replicates for high fold-change DEGs, that three clean replicates per condition are sufficient when designing an RNA-seq experiment with the primary goal of identifying those DEGs with a fold change > 2.0. In this study, we identify differentially expressed genes with a fold change > 2.0 and false discovery rate (FDR) value < 0.05 being set as threshold. It was therefore inferred that the number of samples has no adverse effect on our results. Finally, in previous studies about the transcriptome profiles of the skeletal muscle in sheep (Arora et al., 2019; Cheng et al., 2020), cattle (Cassar-Malek et al., 2017; Zhang et al., 2018) and goats (Lin et al., 2017; Wang et al., 2017; Zhan et al., 2018), the number of samples per condition was less than four.

The sequencing depth of RNA-Seq is an important index for the detection efficiency of transcripts, especially for the low-expression transcripts. Mortazavi et al. (2008) found that a greater sequencing is necessary for accurately detecting and accessing the abundances of the low-expression transcripts. In this study, an average of 89 and 87 million clean reads were produced from samples collected from LC and ZB goats, respectively. This is substantially more than those reported in muscle tissues of Jianzhou Big-Eared goats with 47–55 million clean reads (Lin et al., 2017) and Huanghuai goats with 27 million clean reads (Wang et al., 2016). This suggests that more transcripts, especially for low-expression transcripts were detected in this study. This argument is supported by more expressed genes found in the study compared to the expression of 14,981 genes reported for the muscle tissue of Huanghuai goats (Wang et al., 2016). It could be speculated that more genes found in the study may be mainly arisen from the low-expression genes compared to the observation by Wang et al. (2016).

The mapped rate of clean reads against reference genome is one of the important factors evaluating the quality of sequencing reads. Dobin et al. (2013) suggested that higher mapped rates guaranteed higher sequencing accuracy and the lower genomic variations in RNA-Seq. In the study, the average mapped rate of the clean reads against goat reference genome ARS1 was over 95%, and it was higher than those reported in previous studies of muscle tissues in Jianzhou Big-Eared goats and Huanghuai goats, with a mapped rate of 71.84% (Lin et al., 2017) and 74.52% (Wang et al., 2016), respectively. Once again this demonstrated the accuracy of RNA-Seq data both from LC and ZB goats in the study.

*PEBP1* and *BDNF* were one of the most prominent up-regulated genes in ZB goats with lower carcass weight and intramuscular fat content. *PEBP1* primarily acts as a negative regulator of myogenesis in mammals. The decreased expression of *PEBP1* promoted the postnatal skeletal muscle growth and development in rats (Sun et al., 2009). BDNF is a contraction-induced myokine and increased muscle fat oxidation via an AMPK-dependent manner in mice (Matthews et al., 2009). Zhu et al. (2019) reported that BDNF increased adipose lipolysis by activating sympathetic innervation of adipose tissues and ultimately caused a reduction of body adiposity in mice. It was therefore inferred that there was a similar effect of *BDNF* on adipose lipolysis in goats, resulting in the lower intramuscular fat content in ZB goats.

*ASIP* was the most prominent down-regulated gene in ZB goats with a 1210.1-fold decrease in expression. *ASIP* plays pleiotropic roles in the fat deposition, content of fatty acid in meat and carcass traits. *ASIP* stimulated the activity of fatty acid synthase to induce the lipogenesis by regulating the intracellular concentration of Ca2^+^ (Zemel et al., 2004). Variation in the *ASIP* has been reported to be associated with carcass weight, back fat thickness, percentage of carcass fat coverage, content of linoleic acid and α-linolenic acid in cattle (Albrecht et al., 2013). It has also been reported that the expression level of *ASIP* was significantly up-regulated in beef with higher intramuscular fat content (Albrecht et al., 2012). This finding was in accordance with our observation that *ASIP* was the most prominent down-regulated gene in the *Longissimus dorsi* muscle of ZB goats with lower intramuscular fat content in the study.

Concerning the top five down-regulated genes in the skeletal muscle of ZB goats, we next highlight the LOC102178315 (Heat shock 70 kDa protein 1B-like), a Heat shock 70 kDa protein (Hsp70) family member. Although the role of LOC102178315 (Heat shock 70 kDa protein 1B-like) in muscle growth and development was not well established, Hsp70 protein play important roles in regulating meat tenderness, and also is involved in muscle growth and development. Hsp70 is referred to be a good biomarker of low meat tenderness (Picard et al., 2014). Bernard et al. (2007) found that Hsp70/DNAJA1 complex decrease meat tenderness through its anti-apoptotic role. In addition, it has been reported that the expression level of *Hsp70* was lower (*P* < 0.05) in muscle from suckling goats with tender meat than older goats with tougher meat (Saccà et al., 2019). It was therefore not surprising that the expression level of LOC102178315 was lower in ZB goats with more tender meat. Meanwhile, Hsp70 was involved in regulating myoblast differentiation by interacting with MK2 to stabilize p38MAPK (Fan et al., 2018). As the same as LOC102178315, the role of 60S ribosomal protein L17-like (LOC102186409) in muscle growth and development may be reflected by RPL17 (60S ribosomal protein L17). RPL17 is a core protein of the large ribosomal subunit (60S) and plays key role in protein synthesis (Meng et al., 2010). The skeletal muscle growth and hypertrophy are accompanied by a large amount of protein synthesis. On contrary, skeletal muscle loss is accompanied by small muscle fiber size and low protein content in meat (Cai et al., 2016). It was therefore inferred that the lower expression of LOC102186409 may contribute to lower carcass weight of ZB goats by decreasing protein synthesis to inhibit muscle growth. As another down-regulated gene in ZB goats, the expression of *HEPHL1* has been found to increase myoblast fusion in mice (Lopezjimenez et al., 2010). This suggests that *HEPHL1* may increase carcass weight by promoting myoblast fusion in goats.

As a result of the enrichment analysis, GO terms closely related to muscle growth and development were found herein, including muscle structure development, regulation of muscle system process, myoblast differentiation, muscle cell differentiation and myofibril. These terms were enriched by nine down-regulated genes (*LGALS1*, *SOX8*, *GREM1*, *TNNT2*, *DNER*, *CHRNA3*, *COL3A1*, *SMPX*, and *ATP2B2*) in ZB goats. These down-regulated genes have been reported to be essential for muscle growth and development. For example, *LGALS1* plays important roles in myoblast fusion, skeletal muscle differentiation, regeneration and myotube growth (Watt et al., 2002). As a muscle satellite cell marker, Sox8-deficient mice exhibited a significant reduction in body weight (Sock et al., 2001). *TNNT2* is related to the contraction of striated muscles, and variations on its expression were associated with loin eye area in pigs (Ropka-Molik et al., 2018). In turn, *SMPX* is a regulator of murine skeletal muscle hypertrophy (Kemp et al., 2001).

The GO term extracellular matrix was enriched by nine down-regulated genes (*SPARC*, *COL3A1*, *FBN3*, *CCDC80*, *LOXL2*, *LGALS1*, *LOC102187872*, *FGF1*, and *SPON2*) in ZB goats. The DEGs were also enriched in extracellular matrix in sheep (Miao et al., 2015) and pigs (Xu et al., 2018) with different muscle traits. Extracellular matrix plays dual roles in both myogenesis and adipogenesis. For example, extracellular matrix has a strong effect on muscle cell differentiation, muscle fiber force maintenance and repair (Liu et al., 2018). In addition, it has been reported that extracellular matrix protein was significantly accreted on the surface of differentiated bovine intramuscular adipocytes, suggesting that extracellular matrix is required during the adipocyte differentiation (Nakajima et al., 2002). Of these DEGs, *SPARC*, *CCDC80*, and *LGALS1* have been reported to be involved in the process of myogenesis, while *CCDC80* and *FGF1* have been reported to participate in the adipogenesis. For example, the inhibition of *SPARC* led to a decrease in the diameter of myofibers (Nakamura et al., 2013). *SPARC* also promotes the differentiation of C2C12 myoblasts during the myotube formation (Melouane et al., 2018). *CCDC80* has been reported to have a positive role in human skeletal myotubes differentiation (Raymond et al., 2010). The knockdown of *CCDC80* inhibited mice adipocyte differentiation (Tremblay et al., 2009). *FGF1* has been reported to be required for the differentiation of human preadipocytes (Hutley et al., 2011).

The carcass weight of ZB goats was about half of LC goats in the current study. It was therefore important to investigate some signaling pathways regulating muscle mass. Hippo signaling pathway was one of the most significant pathways enriched by four down-regulated genes (*TEAD4*, *FGF1*, *PPP2R2C*, and *BIRC5*) in ZB goats. Hippo signaling can increase muscle mass by promoting the proliferation of skeletal muscle stem cells, or enhancing the myogenic differentiation of the cells in mice (Gnimassou et al., 2017; Sun et al., 2017). YAP is a core effector of the Hippo pathway. It act as a positive regulator of skeletal muscle fiber size via interaction with TEAD transcription factors, causing an increase of muscle mass (Watt et al., 2015). In addition, *TEAD4*, encoding one of TEAD family members, has been reported to promote myoblast proliferation and differentiation by combining downstream effectors of Hippo signaling pathway (Sun et al., 2017). Furthermore, the expression of *FGF1* was induced in differentiating myoblasts and regenerating mouse muscle, whereas the knockout of *FGF1* repressed myoblast differentiation (Conte et al., 2009), suggesting that *FGF1* may increase muscle hypertrophy by promoting differentiation of muscle cells. In the study, the expression levels of *TEAD4* and *FGF1* were decreased in the skeletal muscle of ZB goats with lower carcass weight. The two genes contribute to interpret the lower carcass weight of ZB goats.

Collagen fibers in the muscle can positively regulates the myogenesis, adipocyte differentiation, and lipid synthesis (Nakajima et al., 2002). Additionally, the muscle collagen content is associated with meat tenderness (Dransfield et al., 2003). According to Nakajima et al. (2002), collagen types I–VI promote the differentiation of bovine intramuscular adipocytes and accelerate lipid synthesis. As might be expected, the collagen family genes *COL3A1*, *COL6A6*, *COL21A1*, and LOC102187872, were down-regulated in ZB goats in the present study. These results may explain why ZB goats have lower carcass weight and intramuscular fat content compared to LC goats.

As one of the most enriched pathways for up-regulated genes in ZB goats, JAK-STAT signaling pathway is activated by various cytokines and regulates the differentiation and proliferation of myoblasts (Jang and Baik, 2013). In the study, four up-regulated genes *CDKN1A*, *SOCS2*, *PIM1*, and *CISH* in ZB goats were enriched in the pathway. *CDKN1A* and *SOCS2* play negative roles in muscle growth and development. CDKN1A can cause C2C12 myoblasts myotube and muscle fiber atrophy by promoting protein breakdown (Ebert, 2012). *SOCS2* was found to inhibit the differentiation of C2C12 cells and myotube formation by reducing the expression levels of myotube differentiation related genes *MyHC*, *MyoD*, and *MyoG*, or elevating the expression of *MSTN* (Ebert, 2012). The higher expression of *CDKN1A* and *SOCS2* were therefore related to lower carcass weight in ZB goats compared to LC goats.

Other DEGs of interest, which might also be contributing to underpin the difference in carcass weight and intramuscular fat content between LC and ZB goats, include up-regulated gene *BTG2* in the *Longissimus dorsi* muscle tissue of ZB goats. *BTG2* has been reported to inhibit the proliferation of primary muscle fibers in pigs (Feng et al., 2007) and suppress lipid accumulation and adipogenesis by down-regulating the JAK2-Stat3 signaling pathway (Kim et al., 2016).

Seven down-regulated genes in ZB goats (*FABP3*, *SPARC*, *RET*, *CRHBP*, *HCAR1*, *SPON2*, and *FNDC5*) were enriched in fat related GO terms, including the response to lipid, long-chain fatty acid binding, and the positive regulation of fat cell differentiation. *FABP3* encodes a fatty acid transport enzyme and plays major role in the absorption and oxidation of long-chain fatty acids during fat synthesis and deposition (Schaap et al., 1999). It has been reported that the expression level of *FABP3* was highly positively correlated (*r* = 0.737) with intramuscular fat content in sheep (Huang et al., 2006). *FNDC5* has been reported to promote the proliferation and induce the differentiation of goat adipose-derived stem cells (Dong et al., 2019).

At last, we shed light on two down-regulated genes in ZB goats, the *MB* and *PRKAG3*. *MB* is mainly expressed in cardiomyocytes and myofibers of oxidative skeletal muscle. The gene was also abundantly expressed in fully differentiated C2C12 myotubes (Kanatous and Mammen, 2010). The expression level of *MB* was significantly increased during C2C12 cell differentiation (Kanatous and Mammen, 2010). These suggest that *MB* may increase muscle mass by enhancing differentiation of muscle cells. *PRKAG3* encodes AMP-activated protein kinase (AMPK) γ3 isoform, which is mainly involved in carbohydrate and lipid metabolism in skeletal muscle. Variations in *PRKAG3* were associated with intramuscular fat content, meat color, pH, water-holding capacity and carcass composition in pork (Yoo et al., 2007).

The water-holding capacity is an important index of meat quality and affects sensorial quality and economical value of meat, which can be well reflected by drip water loss rate. The water-holding capacity is also related to meat juiciness after chewing, and intramuscular fat content. We found that meat from ZB goats with lower intramuscular fat content had higher water-holding capacity than those from LC goats. Daszkiewicz et al. (2005) also confirmed the negative relationship between water-holding capacity and intramuscular fat content in Large White and Landrace pigs. Drip water loss of meat is mainly caused by shrinkage of myofibrils due to a series of post-mortem event, such as apoptosis, energy reserves, oxidative stress, pH, concentration of Ca^2+^ (Huff-Lonergan and Lonergan, 2005; Tang et al., 2019). Of these factors, pH value has been reported to have negatively correlation with drip water loss in meat (Warner et al., 1997). The negative correlation was also confirmed in the study. Two down-regulated genes (*NOS2* and *PRKAG3*), and one up-regulated gene (*IFI6*) identified in ZB goats were associated with pH value and drip water loss rate. The increased expression level of *NOS2* decreased pH at 45 min post-mortem and pH at 24 h post-mortem, but increased drip water loss rate in chicken breast muscle (Tang et al., 2019). Pigs with dominant mutation in *PRKAG3* had a higher glycogen content, lower pH, and reduced water-holding capacity (Milan et al., 2000). On the contrary, the high expression of *IFI6* resulted in a reduction of drip water loss rate by anti-apoptosis functions and by regulating Ca^2+^ (Kayan et al., 2011).

## Conclusion

The study compared the transcriptome profiles of the *Longissimus dorsi* muscle from LC and ZB goats with phenotypic difference in meat production performance. Some important DEGs related to skeletal muscle growth and development (*PEBP1*, *SPARC*, *COL3A1*, *SOCS2*, *BTG2*, and *MB*), intramuscular fat deposition (*FABP3*, *PRKAG3*, and *CCDC80*), meat tenderness (*Hsp70*), muscle water-holding capacity and pH (*PRKAG3* and *NOS2*), were identified. In addition, several GO terms and KEGG pathways closely related to muscle growth and development, and intramuscular fat deposition were found, including muscle structure development, positive regulation of fat cell differentiation, JAK-STAT pathway and Hippo signaling pathway. These DEGs, GO terms and KEGGs, helped us to better understanding the biology behind muscle and fat deposition for the two studied breeds and, in the future the DEGs may be used to improve the accuracy of selection for meat-related traits in goats.

## Data Availability Statement

The original contributions presented in the study are publicly available. This data can be found here: NCBI, GenBank with accession numbers SRR13008213-SRR13008222.

## Ethics Statement

The animal study was reviewed and approved by the Ministry of Science and Technology of the People’s Republic of China. Written informed consent was obtained from the owners for the participation of their animals in this study.

## Author Contributions

JS, JW, and YZL: conceptualization. JS and JW: data curation. JS, JW, and ZH: formal analysis. JW and YZL: funding acquisition. JS and NK: investigation. JH, XL, and SL: project administration. YZL: resources. YS and YJL: software. JW: supervision. LH, LQ, and XW: validation. JS and JW: writing–original draft. JW and YZL: writing – review and editing. All authors contributed to the article and approved the submitted version.

## Conflict of Interest

The authors declare that the research was conducted in the absence of any commercial or financial relationships that could be construed as a potential conflict of interest.

## Footnotes

1 ftp://ftp.ncbi.nlm.nih.gov/genomes/all/GCF/001/704/415/GCF_001704415.1_ARS1

2 http://www.geneontology.org/

## Supplementary Material

Supplementary File 1 The up-regulated genes and down-regulated genes identified in ZB goats compared to LC goats.
